# Insights Gained From Developing Academic-Community Partnerships for Minority Aging, Community-Engaged Research

**DOI:** 10.1093/geroni/igaa057.3095

**Published:** 2020-12-16

**Authors:** Ronica Rooks, Peter Lichtenberg

## Abstract

Increasingly community-engaged research, characterized by collaborations between researchers and community partners, is recognized as an important part of translating research into improved health outcomes and reduced health disparities for community participants. Training community participants to engage in some or all aspects of this research, particularly focusing on racial and ethnic minority older adults, highlights the need to understand its opportunities and challenges. With this symposium we will discuss and reflect on community-engaged and community-based participatory research approaches to community-academic partnerships with minority older adults. The first presentation addresses recruitment, retention, and training of a community advisory board of older African Americans in Michigan. The second presentation addresses a health education outreach and engagement program to improve health outcomes among older African Americans in California. The third presentation combines community engagement with survey design methods for research with older Native Hawaiian and Pacific Islander adults to improve data collection and health outcomes in this U.S. population. The final presentation examines partnerships between a hospital memory clinic, meal delivery service, research university, and low-income health clinic to improve caregiver and dementia patient outcomes for minority older adults. The symposium discussant will address opportunities, challenges, and implications of community-academic partnerships promoting minority aging.

